# Laparoscopic Gastrectomy for Heterotopic Pancreas with Concurrent Gastroduodenal Invagination and Gastric Wall Abscess: A Case Report

**DOI:** 10.70352/scrj.cr.25-0018

**Published:** 2025-04-29

**Authors:** Junki Fukuda, Akira Shibata, Ryosuke Ohashi, Yuma Hane, Takahiro Saito, Kohei Nishigami, Naoto Senmaru, Miri Fujita, Satoshi Hirano

**Affiliations:** 1 Department of Surgery, Steel Memorial Muroran Hospital, Muroran, Hokkaido, Japan; 2 Department of Gastroenterological Surgery II, Hokkaido University Graduate School of Medicine, Sapporo, Hokkaido, Japan; 3 Department of Pathology, Steel Memorial Muroran Hospital, Muroran, Hokkaido, Japan

**Keywords:** gastric heterotopic pancreas, gastroduodenal invagination, gastric wall abscess, laparoscopic surgery

## Abstract

**INTRODUCTION:**

Heterotopic pancreas refers to pancreatic tissue located outside its normal position and lacking anatomical or vascular continuity with the pancreas. Gastric heterotopic pancreas (GHP) is generally asymptomatic, but in rare cases large GHP lesions can cause gastric outlet syndrome or gastroduodenal invagination. GHP may also occasionally cause acute pancreatitis and abscess formation in the gastric wall. This report describes a rare case of GHP with concurrent gastroduodenal invagination and gastric wall abscess treated via laparoscopic distal gastrectomy.

**CASE PRESENTATION:**

A 46-year-old man was admitted to the hospital with abdominal pain and vomiting. Computed tomography revealed a 40-mm low-density mass in the gastric pylorus, and gastroduodenal invagination. Gastroscopy confirmed a submucosal lesion at the gastric pylorus causing pyloric stenosis. The patient underwent laparoscopic distal gastrectomy with Roux-en-Y reconstruction. Histopathological examination revealed a gastric submucosal lesion containing pancreatic tissue with acinar cells and ducts, without islets of Langerhans, leading to a diagnosis of Heinrich type II GHP. The submucosal lesion also contained inflammatory components such as neutrophils and foamy histiocytes, forming a gastric wall abscess. Finally, the patient was discharged on postoperative day 11 and is progressing well 7 months after surgery.

**CONCLUSIONS:**

Herein we report the first case of laparoscopic distal gastrectomy for GHP with concurrent gastroduodenal invagination and gastric wall abscess resulting in a favorable outcome.

## Abbreviations

GHP
: gastric heterotopic pancreas
HP
: heterotopic pancreas

## INTRODUCTION

Heterotopic pancreas (HP) is defined as the presence of pancreatic tissue outside its normal location, and without anatomical and vascular continuity with the main body of the pancreas.^1)^ It is usually asymptomatic and detected incidentally via imaging, but it can become symptomatic when complicated by obstruction, inflammation, bleeding, or malignant transformation. When a gastric HP (GHP) lesion is particularly large, in rare cases it may cause gastric outlet syndrome or gastroduodenal invagination, resulting in abdominal pain and vomiting.^1–10)^ Patients with GHP can also occasionally develop acute pancreatitis leading to abscess formation in the stomach wall.^11–13)^ Herein we report a rare case of laparoscopic distal gastrectomy for GHP with concurrent gastroduodenal invagination and gastric wall abscess.

## CASE PRESENTATION

**Fig. 1:**
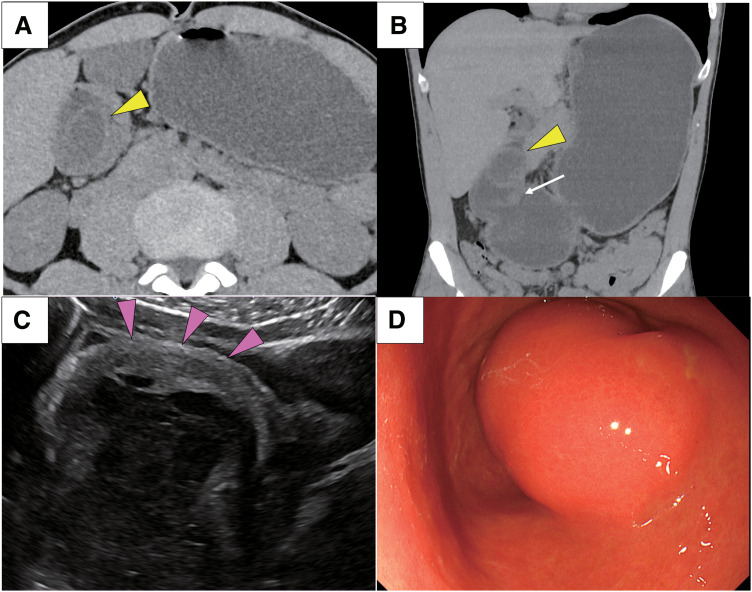
Images of preoperative examinations. (**A, B**) Abdominal computed tomography depicted a 40-mm low-density mass that extended beyond the pyloric ring (white arrow) and descended into the duodenal bulb, showing a target sign (yellow arrowhead). (**C**) Ultrasonography depicted a 40-mm mass with fluid accumulation arising from the gastric submucosa (magenta arrowheads). (**D**) Gastric fiber revealed a submucosal lesion on the posterior wall of the gastric pylorus.

A 46-year-old man presented to the emergency department with abdominal pain and vomiting. He was a heavy drinker, consuming approximately 100 g of alcohol daily, and a heavy smoker, having smoked 20 cigarettes daily for the past 25 years. On physical examination, he exhibited upper quadrant pain and no rebound pain. Laboratory tests revealed elevated inflammatory markers, including white blood cell count 17900/μL and C-reactive protein 1.38 mg/dL. Abdominopelvic computed tomography revealed a 40-mm low-density mass that extended beyond the pyloric ring and descended into the duodenal bulb (**Fig. 1A**, **1B**). Ultrasonography depicted a 40-mm mass with fluid accumulation arising from the gastric submucosa (**Fig. 1C**). The stomach was markedly dilated due to gastroduodenal invagination, thus the patient was urgently admitted to the hospital after placement of a gastric tube. The next day, gastric fiber revealed a submucosal lesion on the posterior wall of the gastric pylorus. Gastroduodenal invagination was relieved by gastric tube decompression, but pyloric stenosis due to a large lesion was still present (**Fig. 1D**). Based on these findings, the preoperative differential diagnosis was submucosal cystic tumor of gastric origin, including malignant lymphoma, carcinoid, gastric cyst, and HP. The patient resumed oral intake once, but due to recurrence of vomiting symptoms we decided on surgical resection. Prior to the operation, the patient was kept on no food or drink, with a daily volume of 2000 mL of supplemental fluids and an intravenous infusion of proton pump inhibitors.

**Fig. 2:**
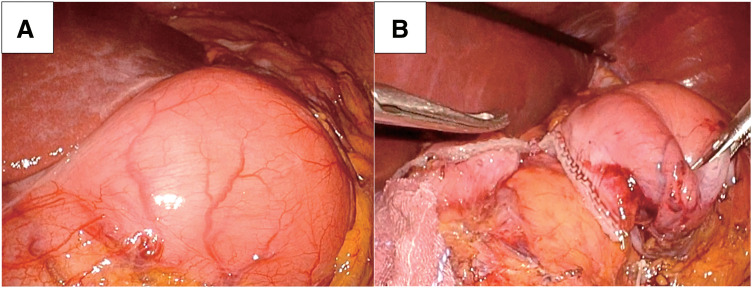
Intraoperative images of laparoscopic surgery. (**A**) Intraoperative findings indicated that the lesion was localized to the gastric pylorus. (**B**) The duodenum on the anorectal side of the lesion was divided using a linear stapler.

Laparoscopic distal gastrectomy with Roux-en-Y reconstruction was performed. Since our institution routinely employs the Roux-en-Y reconstruction for distal gastrectomy to prevent residual gastritis, we consistently opted this reconstruction method in this case. Intraoperative findings indicated that the lesion was localized to the gastric pylorus and the stomach was dilated, although the gastroduodenal invagination had already been released (**Fig. 2A**). The oral side was dissected at the gastric angle, and the anal side margin was dissected at the duodenum using a linear stapler after sufficient margins of the tumor were confirmed grossly from outside the gastric wall (**Fig. 2B**). The operation time was 248 min and blood loss was 20 mL.

**Fig. 3:**
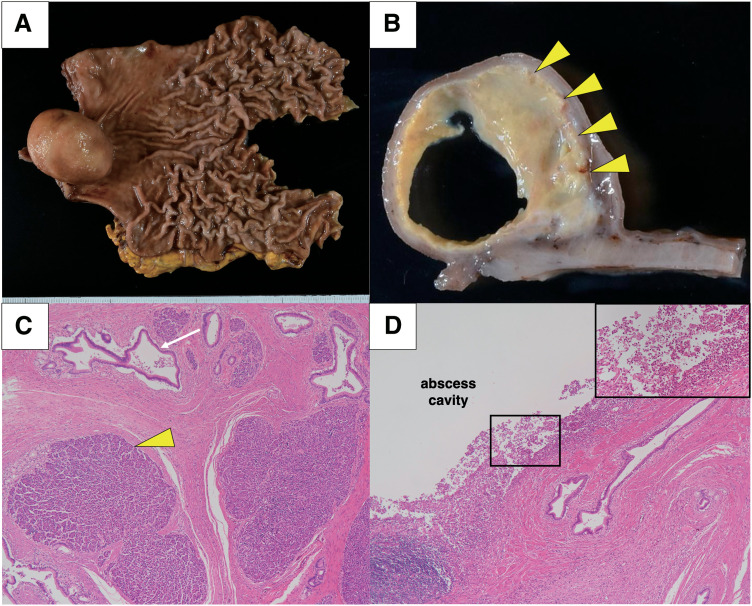
Macro and microscopic findings of the resected specimen. (**A, B**) Surgical specimens indicated a submucosal cystic tumor filled with an abscess (yellow arrowheads) in the gastric pylorus. (**C**) Histopathological examination revealed pancreatic tissue with acinar cells (yellow arrowhead) and pancreatic ducts (white arrow). (**D**) In the heterotopic pancreas, an abscess containing neutrophils and foamy histiocytes (boxed area) was continuously formed from the pancreatic duct tissue. The boxed area is magnified in the inset.

Surgical specimens showed a submucosal cystic tumor at the gastric pylorus, which was filled with an internal abscess (**Fig. 3A**, **3B**). Histopathological examination revealed pancreatic tissue containing acinar cells and pancreatic ducts from the submucosa to the muscularis propria of the stomach without islets of Langerhans, leading to a diagnosis of Heinrich type II GHP^14)^ (**Fig. 3C**). Additionally, abscesses containing neutrophils and foamy histiocytes were continuously formed from the pancreatic duct tissue within the heterotopic pancreas (**Fig. 3D**). The patient resumed oral intake and was discharged on postoperative days 2 and 11, respectively, and is progressing well 7 months after surgery.

## DISCUSSION

HP is defined as pancreatic tissue without substantial anatomical or vascular connection to the pancreas, and was first described by Jean-Schultz in 1729.^15)^ In autopsy studies the incidence of HP generally ranged from 0.5% to 13.0%, and was more common in males aged 30−50 years.^16)^ The most common sites of HP are the duodenum (93.6%), stomach (24%–38%), jejunum (0.5%–27.0%), and Meckel’s diverticulum (2.0%–6.5%).^1)^ The majority of GHP originate from the submucosa and occasionally from the muscularis layer. GHP is usually asymptomatic, but depending on its location and size it can cause abdominal symptoms such as vomiting and pain. HP-related symptoms can be divided into two main categories, those due to the size of the mass and those due to pancreatitis.^17)^

**Table 1** summarizes the details of GHP with gastric outlet syndrome.^1–10)^ We searched PubMed using the keywords “gastric ectopic pancreas” or “gastric outlet syndrome” (1946–2024) and included studies with detailed patient information. The median age of 7 male and 5 female patients was 44 years (range 26–53 years). The most common symptoms were abdominal pain and vomiting. In all 12 cases the lesions were localized to the pylorus, with a median size of 40 mm (range 20–60 mm). Most GHPs are reportedly located within 50 mm of the pylorus.^18)^ Armstrong et al.^19)^ reported a significant correlation between abdominal symptoms and large lesions (>15 mm in diameter). Larger lesions may cause pyloric stenosis due to gastric outlet obstruction, and rarely they coexist with gastroduodenal invagination, such as in the present case. In cases where the lesion is distant from the pyloric ring, laparoscopic surgery, including laparoscopic endoscopic cooperative surgery, has been reported to be a favorable procedure.^20)^ However, in cases of GHP with gastric outlet syndrome the lesion is often located close to the pyloric ring.^1–7)^ Therefore, we opted for antrectomy in the current case in order to avoid postoperative outlet obstruction due to gastric deformity after local resection of the stomach, and to ensure a sufficient surgical margin. Laparotomy was often chosen in previous cases,^1–3,5–9)^ but laparoscopic surgery could also be performed if the stomach was decompressed and a safe surgical field was secured. GHP is one of the differential diagnoses of submucosal cystic tumors of gastric origin, although it is rarely confirmed preoperatively.^21)^ Endoscopic ultrasound-guided fine needle aspiration was not performed in this case because we believed that fluid was the main component inside the cyst and that a definitive diagnosis was unlikely to be obtained. Abdominal ultrasonography also revealed that the tumor originated from the submucosal layer, thereby excluding epithelial tumors such as gastric adenocarcinoma and neuroendocrine tumor. Based on these findings, we concluded that epithelial malignancy was improbable and attempted a gastrectomy without lymph node dissection.

**Table 1:** Summary of previously reported cases of gastric heterotopic pancreas with gastric outlet syndrome

First author (year)	Age/sex	Symptoms	Size (mm)	Invagination	Surgical procedure	Heinrich’s classification	Pancreatitis	Gastric wall abscesses
Huang (2002)^2)^	41/M	Abdominal pain	25	No	Open subtotal gastrectomy	II	No	No
Huang (2002)^2)^	53/M	Abdominal pain	20	No	Open subtotal gastrectomy	ND	No	No
Ikematsu (2003)^3)^	26/F	Abdominal pain, vomiting	50	No	Open distal gastrectomy	I	No	No
Ayantunde (2006)^4)^	48/F	Vomiting	25	No	Laparoscopic anterior gastrectomy	ND	No	No
Christodoulidis (2007)^1)^	40/F	Abdominal pain, vomiting	50	No	Open wedge gastrectomy	I	No	No
Jiang (2008)^5)^	46/F	Abdominal pain, vomiting	20	No	Open distal gastrectomy	II	No	No
Trifan (2012)^6)^	31/M	Abdominal pain, vomiting	35	No	Open distal gastrectomy	I	Yes	No
Iwahashi (2019)^7)^	40/M	Abdominal pain, vomiting	60	Yes	Open distal gastrectomy	III	No	No
Xiong (2020)^8)^	44/F	Abdominal distension	40	No	Open distal gastrectomy	III	No	No
Bejiga (2022)^9)^	45/M	Vomiting	ND	No	Open distal gastrectomy	ND	No	No
Mirzaie (2023)^10)^	43/M	Abdominal pain, vomiting	40	No	Laparoscopic distal gastrectomy	II	No	No
Current case (2025)	46/M	Abdominal pain, vomiting	40	Yes	Laparoscopic distal gastrectomy	II	Yes	Yes

F, female; M, male; ND, not described.

Rarely, HP can form gastric wall abscesses.^11–13)^ There are two factors that cause HP to form abscesses: (1) the pancreatic duct may be blocked by a protein plug resulting in inflammatory changes, or (2) the pancreatic enzymes themselves are activated resulting in acute pancreatitis. In the present case, pathologically there was no protein plug in the pancreatic duct, suggesting that acute pancreatitis due to HP triggered the formation of gastric wall abscesses. Intriguingly, chronic alcohol consumption has been reported to induce pancreatitis in GHP,^10)^ and it may have caused the development of pancreatitis in the present case. Here, we finally performed a gastrectomy on a GHP with an abscess. Even if a diagnosis of GHP with abscess had been made preoperatively, a gastrectomy would have still been indicated. Regarding the treatment of GHP with abscess, antimicrobial therapy alone has been reported to lead to inflammation recurrence; therefore, surgical treatment is recommended.^22,23)^ As an alternative treatment, several cases where endoscopic ultrasound-guided drainage was performed have been reported, although the abscess recurred, ultimately requiring a gastrectomy.^12,24,25)^ In summary, whether the cause of the abdominal symptoms was gastroduodenal invasion or gastric wall abscess, our decision to perform a laparoscopic distal gastrectomy was reasonable, and it was supported by previous reports of favorable outcomes with surgical procedures.

## CONCLUSIONS

We report the first case of laparoscopic distal gastrectomy for GHP with gastroduodenal invasion and gastric wall abscess. The case suggests that surgical resection may be an effective treatment option for GHP with concurrent gastroduodenal invasion and gastric wall abscess.

## ACKNOWLEDGMENTS

The authors thank Editage (www.editage.com) for the English language editing. No financial support was received for this study.

## DECLARATIONS

### Funding

Not applicable.

### Authors’ contributions

JF drafted the manuscript. RO, YH, TS, KN, NS, and SH critically revised the manuscript.

All authors read and approved the final manuscript.

### Availability of data and materials

The datasets supporting the conclusions of this article are included within the article and its additional files.

### Ethics approval and consent to participate

This work does not require ethical considerations or approval. Informed consent to participate in this study was obtained from the patient.

### Consent for publication

The patient provided written informed consent to publish this case report.

### Competing interests

The authors declare that they have no competing interests.
